# Evidence for the Management of Bronchopulmonary Dysplasia in Very Preterm Infants

**DOI:** 10.3390/children8040298

**Published:** 2021-04-13

**Authors:** Tobias Muehlbacher, Dirk Bassler, Manuel B. Bryant

**Affiliations:** Department of Neonatology, University Hospital Zurich, 8091 Zurich, Switzerland; dirk.bassler@usz.ch (D.B.); manuel.bryant@usz.ch (M.B.B.)

**Keywords:** bronchopulmonary dysplasia, chronic lung disease, prevention, treatment, preterm infant

## Abstract

Background: Very preterm birth often results in the development of bronchopulmonary dysplasia (BPD) with an inverse correlation of gestational age and birthweight. This very preterm population is especially exposed to interventions, which affect the development of BPD. Objective: The goal of our review is to summarize the evidence on these daily procedures and provide evidence-based recommendations for the management of BPD. Methods: We conducted a systematic literature research using MEDLINE/PubMed on antenatal corticosteroids, surfactant-replacement therapy, caffeine, ventilation strategies, postnatal corticosteroids, inhaled nitric oxide, inhaled bronchodilators, macrolides, patent ductus arteriosus, fluid management, vitamin A, treatment of pulmonary hypertension and stem cell therapy. Results: Evidence provided by meta-analyses, systematic reviews, randomized controlled trials (RCTs) and large observational studies are summarized as a narrative review. Discussion: There is strong evidence for the use of antenatal corticosteroids, surfactant-replacement therapy, especially in combination with noninvasive ventilation strategies, caffeine and lung-protective ventilation strategies. A more differentiated approach has to be applied to corticosteroid treatment, the management of patent ductus arteriosus (PDA), fluid-intake and vitamin A supplementation, as well as the treatment of BPD-associated pulmonary hypertension. There is no evidence for the routine use of inhaled bronchodilators and prophylactic inhaled nitric oxide. Stem cell therapy is promising, but should be used in RCTs only.

## 1. Introduction

Bronchopulmonary dysplasia (BPD) is one of the most common morbidities in very preterm infants. In 1967, Northway introduced the term “bronchopulmonary dysplasia” for the fibrotic pulmonary remodeling of preterm infants after respiratory distress syndrome [1]. The definition of BPD changed since Northway’s definition of oxygen demand at 28 days of life: first, to the oxygen requirement at 36 weeks postmenstrual age (PMA) [2] and, then, to the “new” (in the cited studies most commonly used) criteria of the National Institutes of Child Health and Human Development (NICHD)/National Heart, Lung, and Blood Institute (NHLBI) workshop of 2001 that was developed with the purpose of identifying affected patients in a more precise way and quantifying the severity of the disease [3]. The latter defines BPD as oxygen demand at 28 days of life and a stratification of severity at 36 weeks PMA by the need for supplemental oxygen and respiratory support (Table 1). However, the definition of BPD is still a matter of ongoing debate regarding the changes in neonatal care during the last decades, e.g., the widespread use of a nasal cannula with different flow settings [4]. The incidence of BPD ranges from 10% to 40%, with an inverse correlation with gestational age and birthweight [5]. As neonatal intensive care substantially improved, the mortality rate of preterm infants decreased; however, the rates of BPD remained basically the same or even increased as more immature infants survived [6,7]. Addressing the change in the pathophysiological mechanism of BPD development, Jobe coined the term “new” BPD for an altered lung development in these extremely preterm infants in contrast to the pre-surfactant lung injury to oxygen toxicity and aggressive mechanical ventilation [8]. A complex mixture of developmental plasticity, inflammation, injury and repair of the immature lungs leads to the clinical entity “BPD” with altered alveolarization and vascularization [9]. It is associated with long-term sequelae as persistent respiratory symptoms, impaired lung function, pulmonary hypertension and adverse neurodevelopmental outcomes and is therefore associated with increased rates of rehospitalization and late mortality [4,5,10,11]. As no “silver bullet” for the prevention or treatment of BPD exists, it is essential to apply a multifactorial approach to the management of BPD in very preterm infants. This review focuses on everyday strategies regarding the management of BPD and on controversially discussed interventions and their impact on developing BPD.

## 2. What Is the Evidence for?

### 2.1. Antenatal Corticosteroids

Prophylactic treatment with antenatal corticosteroids (ANS) to accelerate lung maturation has been first described by Liggins and Howie in a randomized controlled trial (RCT) in 1972 aiming at the severity of respiratory distress syndrome [12]. The effect on an additionally decreased neonatal mortality and other morbidities in a systematic review [13] led to the NIH consensus paper recommending antenatal corticosteroids for impending preterm birth from 24 weeks to 34 weeks of gestation [14]. The most common regimens consist of two doses of intramuscular injection of 12-mg betamethasone 24 h apart or four doses of 6-mg betamethasone every 12 h [15]. A recent meta-analysis included 30 randomized controlled trials with 8158 preterm infants that confirmed the previous findings with a reduction of perinatal death (relative risk (RR) 0.72, 95% confidence interval (CI) 0.58–0.89, *n* = 6729), but no reduction of BPD could be shown (RR 0.86, 95% CI 0.42–1.79, *n* = 818) [16]. A similar result has been shown by large prospective population-based observational studies [17,18,19] and by a meta-analysis of studies involving small for gestational age infants [20]. However, the before mentioned Cochrane review did not evaluate the effects of ANS in extremely preterm infants due to a lack of sufficient data in RCTs. A meta-analysis of observational studies analyzing these particular infants born before 25 weeks of gestation also found a highly significant reduction in mortality (odds ratio (OR) 0.48, 95% CI 0.42–0.55, *n* = 13,443) [21]. The BPD rate was higher in this study (OR 1.32, 95% CI 1.04–1.67, *n* = 7983), but the composite outcome of BPD and death was reduced by the use of ANS (OR 0.58, 95% CI 0.42–0.79, *n* = 11782) [21]. The results reflected the competitive outcomes of mortality and bronchopulmonary dysplasia in extremely preterm infants. Two further population-based studies not included in the meta-analysis showed a higher BPD rate and lower mortality as well but did not report on the composite outcome [22,23]. Others could show a reduction of both BPD and the mortality rates [24,25,26]. The overall efficiency of ANS depends on the optimum timing of the ANS. Exposure to a completed course of ANS with an interval from 24 h up to seven days after the last injection is the most effective strategy [23,24,25,27,28]. An incomplete course or an interval longer than seven days was less efficient, nonetheless resulting in a better outcome than no exposure to ANS [23,25,27,28]. Another Cochrane review analyzed whether (weekly) repeated courses of ANS are beneficial compared to a single course. They could show a reduction of severe respiratory distress syndrome, yet without an effect on the long-term morbidity or mortality [29]. A post-hoc analysis of a multicenter trial showed a higher mortality in infants of mothers treated with three or more courses of ANS [30].

### 2.2. Surfactant Replacement Therapy

The introduction of surfactant replacement therapy in preterm infants with respiratory distress syndrome by Fujiwara et al. 1980 was a game-changing event in neonatal medicine [31]. The regular administration of surfactant in preterm infants led to the change from the “old” to “new” bronchopulmonary dysplasia [8]. Multiple meta-analyses on the RCTs of the 1990s showed a reduction of the overall mortality and, especially, mortality due to respiratory distress syndrome by surfactant replacement therapy [32,33,34,35,36]. However, no effect could be shown on the incidence of BPD, as now more and, especially, more immature babies survived. Different types of surfactant have proven effective in the treatment of respiratory distress syndrome and the reduction of mortality: synthetic surfactants containing surfactant proteins [37] or protein-free formulation [35] and animal-derived surfactants [36]. A meta-analysis could show a slight superiority of animal-derived surfactants compared to protein-free surfactants regarding mortality (RR 0.89, 95% CI 0.79–0.99, *n* = 5413) and, with borderline significance, the combined outcome BPD at 28 days of life or death (RR 0.95, 95% CI 0.91–1.00, *n* = 3811) [38]. However, there was no difference in BPD at 36 weeks of postmenstrual age (PMA) (RR 0.99, 95% CI 0.91–1.09, *n* = 1896) or combined outcome BPD at 36 weeks PMA or death (RR 0.97, 95% CI 0.90–1.04), neither in prevention nor treatment trials [38]. There was also no difference in mortality or BPD in a meta-analysis evaluating animal-derived surfactants versus protein-containing synthetic surfactants [39]. Two meta-analyses on RCTs comparing bovine and porcine surfactants showed conflicting results. Singh et al. reported an inferiority of bovine surfactant compared to poractant with increased rates of mortality (RR 1.44, 95% CI 1.04–2.00, *n* = 901) and combined BPD or mortality (RR 1.30, 95% CI 1.04–1.64) [40]. There was no difference in the rates of BPD at 36 weeks PMA (RR 0.94, 95% CI 0.79–1.12, *n* = 899). In a subgroup analysis at the initial dose of poractant (≤ 100 mg/kg versus > 100 mg/kg), these results remained only in the group with the (recommended) higher initial dose of poractant [40]. In contrast, Sánchez-Luna et al. included more recent RCTs and found no difference in mortality (OR 1.35, 95% CI 0.98–1.86) or BPD at 36 weeks PMA (OR 1.25, 95% CI 0.96–1.62), but no data on the combined outcome BPD or death has been given in this meta-analysis [41]. They also addressed the question about the recommended dosage regimen: Trials with 100 mg/kg for beractant and poractant showed no differences in BPD or mortality [41,42,43,44,45,46,47]. Trials using the recommended dosage of 100 mg/kg beractant versus 200 mg/kg poractant for the initial dose, followed by 100 mg/kg for subsequent doses, showed no difference in mortality (OR 1.39, 95% CI 0.81–2.38) but a borderline significance of BPD at 36 weeks PMA in favor of poractant (OR 1.34, 95% CI 1.00–1.79) [41].

Not alone, surfactant replacement therapy but, especially, the way of administration and respiratory management around surfactant application have an impact on the development of BPD. The routine application of continuous positive airway pressure (CPAP) and more restrictive use of mechanical ventilation, as described in the section Ventilation Strategies, led to different surfactant administrations. A meta-analysis on INtubation—Surfactant–Extubation (INSURE) versus nasal CPAP without surfactant showed only a trend without significance favoring INSURE regarding mortality (RR 0.94, 95% CI 0.67–1.32), BPD (RR 0.86, 95% CI, 0.71–1.03) and combined BPD or death (RR 0.88, 95% CI, 0.76–1.02) with a moderate level of evidence [48]. One recent randomized controlled trial extended the INSURE procedure by a recruitment maneuver (REC) with high-frequency oscillation ventilation before surfactant administration (IN-REC-SUR-E) [49]. Compared to INSURE, fewer infants treated with IN-REC-SUR-E needed mechanical ventilation during the first 72 h of life; however, there was no difference in the incidence of moderate-to-severe BPD or (per-protocol) mortality [49].

In order to avoid any mechanical ventilation, surfactant administration via a small catheter in spontaneously breathing infants has been established, mostly named as “less-invasive surfactant administration (LISA)” or “minimally invasive surfactant therapy (MIST)”. Consistent results were presented by three meta-analyses favoring LISA/MIST compared to the invasive application of surfactants with a significant reduction in BPD with an OR ranging from 0.47 to 0.72 [50,51,52] and combined BPD or death (OR ranging from 0.74 to 0.75) [50,51]. A network meta-analysis of Isayama et al. found LISA to be the best strategy for noninvasive respiratory management [53]. Compared to a surfactant with continued mechanical ventilation, LISA has a reduced combined rate of BPD or death (OR 0.49, 95% CI 0.30–0.79 and BPD (OR 0.53, 95% CI 0.27–0.96), with the quality of evidence graded as moderate [53]. Another more recent network meta-analysis of Bellos et al., including RCTs and observational studies, could show the superiority of LISA/MIST compared to INSURE regarding both the mortality (OR 0.64, 95% CI 0.54–0.76) and BPD (OR 0.57, 95% CI 0.44–0.73) [54]. The subgroup analysis of infants born before 28 weeks of gestation did not report on BPD but showed the feasibility and efficacy of LISA/MIST in this particularly vulnerable population, with a reduction in mortality (OR 0.56, 95% CI 0.46–0.67) [54].

### 2.3. Caffeine

Caffeine is a methylxanthine that acts as a nonspecific inhibitor of adenosine receptors. There are four different adenosine receptors, and caffeine inhibits three of them (A_1_, A_2A_ and A_2B_) [55,56]. Adenosine is a purine nucleoside that is produced naturally in different human tissues [57]. Caffeine stimulates breathing by various mechanisms. Among others, it has an effect on the diaphragm, minute ventilation and sensitivity to carbon dioxide (CO_2_) sensitivity. It is thanks to the efforts of the Caffeine for Apnea of Prematurity (CAP) Trial Group headed by Barbara Schmidt that we have high-quality and reliable data not only on short-term but also long-term outcomes of caffeine use in extremely preterm infants [58].

CAP was launched to determine whether survival without neurodevelopmental disability at a corrected age of 18 months is improved if apnea of prematurity is managed without caffeine in infants at a high risk of apneic attacks [58]. However, the trial confirmed that caffeine has another clinically important benefit, a reduction in the risk of BPD: 350 out of 963 infants assigned caffeine (36.3%) received supplemental oxygen at 36 weeks PMA, as compared with 447 out of 954 infants (46.9%) assigned a placebo (OR 0.63, 95% CI 0.52–0.76) [58].

The median age of starting study medication in the CAP trial was three days, and the median age of stopping caffeine was 34.4 weeks PMA [58]. Neonatologists asked the question of whether caffeine started earlier than three days, and given longer than 35 weeks, PMA further improves health outcomes. The European consensus guidelines on the management of respiratory distress syndrome recommend that “early caffeine should be considered for babies at high risk of needing mechanical ventilation such as those on non-invasive respiratory support” [59], and this recommendation is supported by various observational studies and systematic reviews [60,61]. Several recent meta-analyses on high versus standard maintenance doses of caffeine came to similar conclusions that high-dose caffeine citrate (10–20 mg/kg per day) might reduce the incidence of BPD, but no general recommendation can be given due to the low quality of evidence [62,63,64,65]. The use of therapeutic drug monitoring of caffeine in routine practice could improve its effects on chronic lung disease and avoid the side effects of high doses [66,67].

### 2.4. Ventilation Strategies

Mechanical ventilation in animals can induce structural and inflammatory changes that mimic human BPD [68,69]. There is a strong link between the use of supplemental oxygen and mechanical ventilation and the incidence of BPD [5]. There are few doubts, that mechanical ventilation, as well as the use of oxygen, are both the risk indicator and risk factor in the pathogenesis of BPD [9]. Therefore, the first and most important advice for mechanical ventilation to prevent BPD is: do not do it [70]. However, a solid number of infants are still unable to survive without mechanical ventilation, and despite a substantial number of RCTs in ventilation strategies, there is uncertainty about how to ventilate in the least harmful manner [71].

Cochrane reviews cover the topics of synchronization [72], elective high-frequency oscillation (HFO) [73], high-frequency jet ventilation (HFJV) [74,75], volume-targeted ventilation [76], positive end-expiratory pressure (PEEP) application [77], inspiratory times [78], permissive hypercapnia [79], neurally adjusted ventilatory assist [80] and the type of trigger that is used for synchronization [81].

#### 2.4.1. High-Frequency Ventilation

High-frequency ventilation therapy is using much smaller tidal volumes than conventional ventilation. Considering volutrauma as a major contributing factor to ventilator-induced lung injury, minimizing tidal volumes is a promising strategy. A Cochrane review summarized the results of 19 RCTs on elective high-frequency oscillation ventilation (HFOV). The combined outcome death or BPD at 36 weeks PMA was reduced with HFOV (RR 0.90, 95% CI 0.84–0.97, *n* = 3329), resulting mainly from a reduction in BPD (RR 0.86, 95% CI 0.78–0.96, *n* = 2786) but with significant heterogeneity between studies. However, using a random-effects model, the reduction of BPD and BPD or death remained significant [73]. The meta-analysis showed an increased incidence of pulmonary air leak (RR 1.19, 95% CI 1.05–1.34), likely due to higher mean airway pressures applied during HFOV [73].

As a consequence of the previous version of this Cochrane review [82], which was criticized because of the heterogeneity of the patient characteristics, ventilators and ventilation strategies used, the authors conducted a patient meta-analysis including 3329 participants from 10 RCTs for which individual patient data were available [83]. The risk of death or BPD at 36 weeks PMA with HFOV was 0.95 (95% CI 0.88–1.03). No subgroup of infants, ventilator type or strategy could be linked to a stronger treatment effect. However, only one of these 10 RCTs used a volume-targeted ventilation mode in the conventional treatment arm. Modern respirators have substantially improved regarding synchronization and tidal volume measurements; therefore, there is a need for RCTs comparing volume-targeted ventilation modes and HFOV [84].

Evidence of high-frequency jet ventilation (HFJV) in very preterm infants is very scarce. A Cochrane review comparing HFOV with HFJV was empty, and a meta-analysis for HFJV versus conventional found only one RCT conducted in the 1980s [85]; therefore, the routine application of HFJV cannot be recommended.

#### 2.4.2. Volume-Targeted Ventilation

Ventilator-induced injury happens mainly in the high-volume injury zone (overinflation, volutrauma) and low-volume injury zone (collapse, atelectotrauma). Animal studies showed that even a few overinflations can cause relevant lung injury [68] and high tidal volumes (volutrauma) are more dangerous than high pressures (barotrauma) [86]. A volume-controlled ventilation uses a constant flow, resulting in a steadily rising (and potentially very high) pressure until the set volume is administered, and the peak pressure is only achieved at the very end of the inspiration. Volume-targeted ventilation (VTV) differs substantially from volume-controlled ventilation and combines control over the pressure and tidal volume. Software algorithms adapt the applied pressure (to a set maximum peak pressure) to the exhaled tidal volumes of the last couple of inflations in order to achieve an expiratory tidal volume matching the set volume as closely as possible [87]. Targeted tidal volumes are usually set to 4–6 mL/kg in conventional ventilation [87] and 1 to 2 mL/kg for HFOV [88].

A meta-analysis of the most recent Cochrane review covered 20 RCTs with 977 infants in 16 parallel group trials, resulting in a reduction of BPD at 36 weeks (RR 0.68, 95% CI 0.53–0.88, *n* = 620) and BPD or death (RR 0.73, 95% CI 0.59–0.89, *n* = 584) [76]. A subgroup-analysis of extremely low birthweight (ELBW) infants did not show significant results regarding BPD (RR 0.81, 95% CI 0.59–1.12, *n* = 202) and combined BPD or death (RR 0.79, 95% CI 0.62–1.01, *n* = 224) [76]. No adverse outcomes were associated with VTV. In contrast, the meta-analysis showed the benefits of VTV in a shorter mechanical ventilation, lower pneumothorax rate, lower rates of intracranial abnormalities and less hypocarbic episodes. An earlier meta-analysis including 18 RCTs came to a very similar result [89]. Since then, a small RCT has been published in Chinese that reports improved short-term outcomes with volume-guarantee ventilation in the weaning phase but no impact on BPD [90].

#### 2.4.3. Permissive Hypercapnia

Reducing the parameters of mechanical ventilation in order to allow arterial pCO_2_ to rise above 45 mmHg is considered “permissive hypercapnia”. The idea behind permissive hypercapnia is, if it is not avoidable, at least to decrease the intensity of mechanical ventilation and to measure the decreased intensity not by the method used but by its effect: a decreased elimination of CO_2_ [91]. However, sudden changes in pCO_2_ should be avoided, as it rapidly alters the cerebral blood flow and increases the risk for cerebral hemorrhage [92]. To gain an adequate magnitude of ventilator adjustment, the protocol of the “Permissive hypercapnia in extremely low birthweight infants” (PHELBI) trial suggested a pragmatic approach by changing the settings by the same relative magnitude as the PaCO_2_ deviation from the target value (e.g., actual PaCO_2_ 40 mmHg and target PaCO_2_ > 50 mmHg = 20% off-target → adjustment: a decreased positive inspiratory pressure (PIP) by 20%) [93].

A Cochrane review included two RCTs, of which one RCT was very small and the other RCT achieved only a 4-mmHg difference between treatment arms [79]. There was no significant difference for death or BPD at 36 weeks PMA, nor for any other relevant outcome. This review has not been updated since 2001. One subsequent RCT randomized infants between 23 and 28 weeks to either normocapnia (35–45 mmHg) or hypercapnia (55–65 mmHg) but was terminated early for failure to achieve the desired CO_2_ difference in the treatment arms. There were no significant differences in BPD at 36 weeks PMA or combined BPD or death but an increase in death or mental impairment in the hypercapnia group (secondary outcome) [94]. The largest RCT (PHELBI trial) was also terminated early because of very slow recruitment and a lack of funding for further extension [93]. Three hundred and fifty-nine infants between 23 and 29 weeks were randomized within the first day of life. Both arms had pCO_2_ targets that were rising on day 4 and day 7 by 5 mmHg, ending in 50–60 mmHg in the low versus 65–75 mmHg in the high pCO_2_ arm. Therefore, in fact, both groups targeted the hypercapnic pCO_2_ levels. Although this study was able to achieve the preset pCO_2_ levels between treatment arms, there was no difference in the primary outcome death or BPD at 36 weeks. However, no adverse effect of hypercapnia on the neurodevelopmental outcome was reported [95].

#### 2.4.4. Supplemental Oxygen

Exposure to oxygen has been associated with BPD. Infants < 30 weeks randomized to SpO_2_ target 95–98% have a higher risk of requiring supplemental oxygen at 36 weeks than infants randomized to 92–94% (OR 1.40, 95% CI 1.15–1.70) [96]. A meta-analysis of the subsequent RCTs demonstrated a lower rate of supplemental oxygen in infants randomized to a SpO_2_ target of 85–89% as opposed to 91–95% (RR 0.81, 95% CI 0.74–0.90), but this was counteracted by an increased risk of death and necrotizing enterocolitis (NEC) [97]. More recently, ventilators have been equipped with pulse oximetry and algorithms to adapt the inspired fraction of oxygen to the set SpO_2_ target. It has been established that these algorithms are able to increase the time spent within the target range and reduce the time at hypoxia and hyperoxia [98]. However, it is unclear whether these improvements translate into a reduction of BPD or other patient-relevant outcomes.

#### 2.4.5. Synchronization

Synchronization, either by higher respiratory rates (60–80/min) as compared to lower rates (30–40/min) or by different triggered ventilation modes, has advantages on the incidence of air leaks but has no established effect on BPD at 36 weeks [72]. The same applies for a meta-analysis of RCTs comparing long with short inspiratory times—there was an advantage for a short inspiration time and higher frequency in terms of air leaks but not in terms of BPD [78]. A Cochrane review [80] identified one RCT on NAVA versus pressure-controlled ventilation, which found no difference in the rates of BPD or other relevant outcomes [99].

#### 2.4.6. Early Extubation

If intubation and mechanical ventilation was either necessary as a life-saving procedure or deemed the least harmful strategy of respiratory support in a particular situation, it does not mean that it needs to be continued until successful extubation can be safely predicted. Preterm infants probably benefit from early extubation [100], even if no pre-extubation test can predict if reintubation will be necessary or not [101,102,103]. Extubation to noninvasive ventilation and caffeine will increase the chances of success, while corticosteroids should be used judiciously [104].

#### 2.4.7. Tracheostomy Placement

Ongoing intensive respiratory support, e.g., mechanical ventilation or high-level noninvasive respiratory support, in infants with established severe BPD at around term-equivalent age prompts the question for further respiratory management. Retrospective cross-sectional studies have shown an increased placement of tracheostomy in very preterm infants in US hospitals over the last decades [105,106], while data from the Neonatal Research Network of Japan have shown a steady rate of interventions [107]. The evidence on tracheostomy is based on retrospective cohorts or case-control studies only. DeMauro et al. evaluated the outcome of infants born before 30 weeks of gestation surviving to a PMA of 36 weeks (*n* = 8683) [108]. Those with tracheostomy (*n* = 304) compared to those without had a higher risk for the composite outcome of death or neurodevelopmental impairment (adjusted OR 3.3, 95% CI 2.4–4.6) but with a lower mortality rate (OR 0.4, 95% CI 0.3–0.7). The authors of the study interpreted the results more likely to be a noncausal association of tracheostomy and neurodevelopmental impairment in these infants [108]. The study also analyzed the impact of timing of tracheostomy on the composite outcome, favoring earlier tracheostomy before 120 days of life in contrast to later tracheostomy (OR 0.5, 95% CI 0.3–0.9). A small single center study included 72 infants born before 32 weeks of gestation or birth weight < 1500 g receiving tracheostomy due to severe BPD (median 51.8 weeks PMA, median 183 days of life) analyzing the growth parameters and participation in developmental therapy four weeks before tracheostomy placement and a four-week period after [109]. Infants showed a significantly higher weekly gain of weight, length and head circumference after tracheostomy despite a lower caloric intake. Additionally, after tracheostomy placement, infants had a lower daily sedation requirement and participated more often in developmental therapies, with a shift of focus from activities promoting physiologic stability to activities promoting developmental skill acquisition [109].

Longitudinal studies on the five-year survival rates and decannulation rates of infants with tracheostomy due to ventilator-dependent BPD show a post-discharge survival of 73–81% and a five-year decannulation rate ranging between 0% to 97%, depending especially on neurological comorbidities [110,111,112]. The median age at decannulation was 32 to 38 months in the cited studies [110,111,112].

### 2.5. Postnatal Corticosteroids

Inflammation, e.g., due to ventilator-induced lung injury, oxygen toxicity or infection, contributes mainly to the development of BPD; therefore, the use of corticosteroids (CS) with a potent anti-inflammatory effect is deemed reasonable. The very widespread use during the 1980s resulted in increasing rates of adverse neurodevelopmental outcome (NDO), especially cerebral palsy. That resulted in an almost complete cessation of postnatal corticosteroid treatment. A (meanwhile updated) meta-regression of Doyle et al. showed the necessity of risk–benefit assessment, as infants with very high risk might profit from CS treatment, even regarding the NDO [113]. Two Cochrane reviews exist that evaluated early (≤seven days) and late (>seven days) CS for the prevention of BPD with a moderate-to-high quality of evidence that both resulted in a reduction of BPD and BPD or death and prevented extubation failure. The relevant side effects included an elevated risk for intestinal perforation, gastrointestinal bleeding, risk of infections, hyperglycemia, hypertension and hypertrophic cardiomyopathy; therefore, the authors concluded that the benefits may not outweigh the adverse effects [114,115]. To reduce BPD, the most efficient CS is dexamethasone, especially in a high-dose regimen (OR 0.29, 95% confidence interval 0.14–0.52, *n* = 659) analyzed by a network meta-analysis but also has the highest risk for cerebral paresis (OR 2.02, 95% confidence interval 0.60–4.67, *n* = 307) [116]. Hydrocortisone might have fewer side effects, especially regarding NDO. A recent individual patient data meta-analysis examined the use of low-dose hydrocortisone as a prophylaxis for early adrenal insufficiency for 10 to 15 days. Treated infants had a higher rate of survival without BPD (OR 1.45, 95% CI, 1.11–1.90, *n* = 979) and a reduction of BPD (OR 0.73, 95% CI 0.54–0.98, *n* = 802) without a difference in death or impaired NDO [117]. However, the latest SToP-BPD RCT randomized 372 infants in a 22-day course of hydrocortisone or placebo and failed to show an effect on the BPD or death at 36 weeks PMA [118], and long-term data are not yet available.

To avoid systemic side effects, topic treatment with CS intratracheal instillation with surfactant as the vehicle and inhaled CS have been used. The meta-analysis of two trials of intratracheal instillation CS show a reduction of BPD (RR 0.19, 95% CI 0.10–0.28, *n* = 381) and the combined outcome of BPD or death (RR 0.26, 95% CI 0.16–0.35, *n* = 381) [119]; yet, data on long-term outcomes are not yet published, and further trials are underway. A meta-analysis of 16 RCTs showed that the inhalation of CS not only reduced the used amount of systemic CS but also led to a reduction of BPD (RR = 0.77, 95% CI 0.65–0.91, *n* = 1168) and BPD or death (RR 0.86, 95% CI 0.75–0.99, *n* = 1285) [120]. No significant change in mortality (RR 0.97, 95% CI 0.42–2.2, *n* = 1270) could be detected. This is of importance, as the mortality rate of the major contributing NEurOSIS study (*n* = 863) was (nonsignificant, but nonetheless) elevated (1.24; 95% CI, 0.91–1.69) [121]. A long-term follow-up of this study showed no difference in NDO but still an increased mortality rate in the budesonide group (RR 1.37, 95% CI, 1.01–1.86) [122]. The authors of NEurOSIS did not find a plausible explanation for the elevated mortality. A meta-analysis of Zheng et al. on long-term effects of the intratracheal administration of CS, including five trials (*n* = 1515), did not find a significant difference in the mortality (RR 1.13, 95 CI 0.90–1.41, *n* = 1465) but, also, neither in the benefits regarding NDO nor adverse effects [123].

### 2.6. Inhaled Nitric Oxide (iNO)

For the purpose of this review, only the prophylactic use of iNO to prevent BPD will be examined.

Endogenous NO is required for alveolar and vascular development [124]. Recent data suggest that there is a relevant vascular component in the development of BPD [125]. There is an association between disturbed NO signaling in BPD; however, it is controversial whether this causal for resulting from developing pulmonary damage or both [126].

In 2011, the results of an NIH Consensus Development Conference and of an individual patient-data meta-analysis have been published. Both concluded that, despite the sound and plausible pathophysiologic basis, there was no benefit nor harm from prophylactic treatment of preterm infants with iNO [127,128]. At that time, it remained unanswered if a particular subgroup of infants might benefit from iNO or if there were treatment-relevant (timing, dose and duration) variables that might explain the gap between basic research and clinical trials. Data from 12 RCTs were analyzed and yielded in a RR of 0.96 (95% CI 0.92–1.01, *n* = 3298) for the primary combined outcome of death (RR 1.05, 95% CI 0.93–1.18) or BPD at 36 weeks PMA (RR 0.93, 95% CI 0.87–1.00). Subgroup analyses indicated a possible benefit for black infants and for trials with starting dosage >5 ppm [127]. Another meta-analysis, on which the NIH Consensus Statement was based, found a borderline significant effect from 14 RCTs for the combined outcome of death or BPD (RR 0.93, 95% CI 0.86–0.99, *n* = 3129) [129,130].

The most recent Cochrane Review included data from 17 RCTs, including three new RCTs since 2011 [131]. Due to significant heterogeneity in the inclusion criteria, they did not perform an overall analysis but divided the trials into three groups: eight trials of early rescue treatment based on oxygenation, four trials examining early routine use of iNO in infants with pulmonary disease and three trials with later start of treatment (>3 days) based on risk of BPD. Risk of death or BPD was similar in all three group of trials (early rescue: RR 0.94, 95% CI 0.87–1.01, *n* = 958; early prophylactic: RR 0.94, 95% CI 0.87–1.02, *n* = 1924 and late: RR 0.92, 95% CI 0.85–1.01, *n* = 1075). A nonsignificant increase of severe intraventricular hemorrhage was found only in the early rescue group (RR 1.20, 95% CI 0.98–1.47, *n* = 773), potentially reflecting a side effect due to the inhibition of platelet aggregation by iNO. One additional RCT, including 451 infants born < 30 weeks of gestation, has been published. There was no difference in survival without BPD at 36 weeks (OR 1.17, 95% CI 0.79–1.73) or BPD severity or IVH [132].

To answer the question whether iNO might be beneficial for infants of African American descent treated with doses >5 ppm and for >seven days, an individual patient data meta-analysis from three RCTs was carried out [133]. It demonstrated a reduced risk for death or BPD (RR 0.77, 95% CI 0.65–0.91) and for BPD alone (RR 0.75, 95% CI 0.61–0.91), no effect at all for White, non-Hispanic and an intermediate nonsignificant effect for Hispanic infants [133].

### 2.7. Inhaled Bronchodilators

Inhaled bronchodilators include nonspecific beta-adrenergic agents, such as isoproterenol, and specific beta-adrenergic agents, such as albuterol, metaproterenol, terbutaline and isoetharine or inhaled anticholinergic agents as atropine or ipratropium. The use of bronchodilators in BPD has been justified by their potential effect of dilating small airways that have muscular hypertrophy. Furthermore, BPD is associated with an increased airway resistance, decreased dynamic compliance and wheezing. If bronchodilators were administered in short-term studies of pulmonary mechanics in infants with BPD, an increased compliance, as well as decreased pulmonary resistance, have been documented [134,135,136]. However, data on clinically relevant outcomes are sparse.

The most recently updated systematic review from the Cochrane Collaboration included only two small studies that reported on 225 infants [137]. One study included 73 infants but reported on 52 infants and examined the prevention of BPD with the use of aminophylline [138]. Methodological quality was reported to be very low according to the GRADE classification system. The use of prophylactic aminophylline was associated with a reduction in BPD at 28 days of life (RR 0.18, 95% CI 0.04–0.74) and a shorter duration of supplementary oxygen therapy. There was no difference in mortality (RR 3.0, 95% CI 0.33–26.99). The second study included in the Cochrane review enrolled 173 infants to look at the prevention of BPD with the use of salbutamol [139]. According to GRADE, the quality of the evidence was moderate. Infants treated with inhaled prophylactic salbutamol compared to controls showed no differences in mortality (RR 1.08, 95% CI 0.50–2.31) nor in BPD (RR 1.03, 95% CI 0.78–1.37) or other complications associated with preterm birth. The study did not report on side effects due to salbutamol. The authors of the systematic review from the Cochrane Collaboration found no eligible trial that studied the use of bronchodilator therapy for the treatment of individuals with BPD [137]. Another systematic review used less strict inclusion criteria and included five further small RCTs (*n* = 84) on beta-agonists [140]. However, due to substantial heterogeneity, no meta-analysis could be performed, and the included studies did only report on short-term measurements with improvement of compliance and resistance but not on clinically relevant outcomes such as BPD or mortality [140]. Despite missing evidence, a recent retrospective multicenter cohort study including 4986 preterm infants born before 32 weeks of gestation with developing BPD at 28 days of life showed the widespread use of inhaled bronchodilators in US hospitals [141]. Twenty-five percent (1224/4986) received at least one dose of inhaled bronchodilators between 28 days of life and discharge or death and, those developing severe BPD at 36 weeks PMA, even 48% (664/1390).

### 2.8. Macrolides

*Ureaplasma* spp. are amongst the most common microbes isolated from maternal samples, as well as neonatal tracheal aspirates [142], and they might cause a chronic pulmonary inflammation contributing to the development of BPD [143,144,145]—in particular, in ventilated infants [146]. A meta-analysis quantified the OR for BPD at 36 weeks to 2.22 (95% CI 1.42–3.47) for *Ureaplasma*-positive infants [147]. Macrolides have a role in pediatric chronic inflammatory pulmonary conditions like cystic fibrosis, so they also might work to prevent BPD, either by an intrinsic anti-inflammatory effect or by affecting colonization status with pathogenic bacteria [148].

Erythromycin was evaluated by two small RCTs, which are summarized but not combined in a meta-analysis in the relevant Cochrane review [149]. Both were randomized infants < 30 weeks, one with unknown and one with positive colonization status. Neither showed a significant difference between Erythromycin and no therapy with regard to death, BPD or the combined. A later meta-analysis evaluated all RCTs on Erythromycin, Clarithromycin and Azithromycin [150]. They found a borderline nonsignificant effect of prophylactic macrolides on the combined death or BPD at 36 weeks (RR 0.89, 95% CI 0.79–1.01, *n* = 438) and a stronger but wider effect in *Ureaplasma*-positive infants only (RR 0.41, 95% CI 0.05–3.13, *n* = 150), with only two studies having a satisfying quality. A subgroup analysis on prophylactic Azithromycin only could show a reduction of BPD (RR 0.83, 95% CI 0.71–0.97, *n* = 310) and combined outcome of BPD or death (RR 0.86, 95% CI 0.77–0.97, *n* = 363) [150]. However, the most recent meta-analysis for Azithromycin including five RCTs with 564 moderate to extremely preterm infants found no difference in BPD, mortality or the combined outcome BPD or death despite the lower duration of supplemental oxygen [151].

The protocol of the AZTEC trial was published recently [152]; it will randomize 796 infants < 30 weeks to a 10-day course of azithromycin. The trial will also investigate the incidence of NEC and of resistance to Azithromycin in stool samples, but it will not be powered to detect the impact of shifts in the microbiome and antimicrobial patterns.

### 2.9. Microbiome and Antibiotic Treatment

The complex interaction of microbiome of intestines and lungs with the developing immune system and organ development will likely provide further insight in the development of BPD [153]. Microbial dysbiosis may be associated with BPD progression and severity [145]. A prolonged antibiotic treatment in infants without culture-positive sepsis and negative inflammatory parameters might lead to such microbial dysbiosis. Higher rates of antibiotic use in culture-negative sepsis were associated with adverse neonatal outcomes, including the development of BPD [154]. However, the risk/benefit ratio of antibiotic use regarding the higher incidence of early- and late-onset sepsis in VLBW and ELBW infants remains challenging; therefore, the implementation of antibiotic stewardship to avoid unnecessary antibiotic treatment is recommended [155].

### 2.10. Patent Ductus Arteriosus

A patent ductus arteriosus (PDA) is a very common morbidity in preterm infants. In extremely preterm infants ≤ 28 weeks of gestation, an incidence of hemodynamically significant PDA of 57% has been reported after the first week with an inverse correlation to gestational age, reaching a maximum of 93% in infants of 23–24 weeks of gestation [156]. The effect of an existing PDA on mortality and morbidities and the resulting urge to treat a PDA are still a matter of debate [157,158]. A meta-analysis of observational studies found major morbidities as intraventricular hemorrhage (IVH), necrotizing enterocolitis (NEC) and BPD related with PDA [159]. These results have to be interpreted with caution, as this association is not necessarily a causality. A meta-analysis of placebo-controlled RCTs could not show any difference in mortality, IVH, NEC or BPD [160]. However, with a median 52% rate of rescue treatment in the control groups, the investigators of the meta-analysis questioned the evidence for good reason [160]. Most RCTs aim at the efficacy to close the PDA and may be underpowered to estimate the impact on neonatal mortality or major morbidities [158]. Therefore, all Cochrane meta-analyses on the different pharmacological treatment options (indomethacin, ibuprofen or paracetamol (acetaminophen) targeting cyclooxygenase-I) failed to show an effect on mortality or BPD despite being effective in PDA closure [161,162,163,164,165]. A recent network meta-analysis evaluated RCTs regarding the different pharmacological interventions (indomethacin, ibuprofen, paracetamol (acetaminophen) or placebo) and found no difference in mortality or morbidities even compared to the placebo but higher rates of side effects as oliguria, especially in indomethacin and ibuprofen [166]. The same conclusion was drawn in another network meta-analysis on RCTs and observational studies [167].

The rates of surgical ligation of the PDA decreased substantially in a US cohort of 61,520 preterm infants born between 23 and 30 weeks of gestation from 8.4% in 2006 to 2.9% in 2015 [168]. A meta-analysis of one RCT and 39 cohort studies evaluating surgical ligation showed a reduced mortality (pooled adjusted OR (aOR) 0.54, 95% CI 0.38–0.77, *n* = 7159), but an increase in morbidities such as BPD (pooled aOR 2.51, 95% CI 1.98–3.18, *n* = 6703) and higher rates of impaired neurodevelopmental outcome (aOR 1.54, 95% CI 1.01–2.33) [169]. The potential survival bias was addressed in a retrospective cohort study including 754 infants adjusting not only for pre- and perinatal confounding factors but, also, for postnatal factors representing the severity of illness [170]. There was still a reduction in mortality (aOR 0.09, 95% CI 0.04–0.21) but no more difference in BPD or the neurodevelopmental outcome [170]. Despite these results, the substantial rates of procedural complications up 44%, including post-ligation cardiac syndrome, vocal cord paralysis and acute kidney injury, have to be taken into account [157].

Percutaneous catheter intervention to close the PDA is associated with high rates of adverse events (23%) and clinically significant adverse events (10%) in a meta-analysis of observational studies on infants of < one year [171]. A newer device has been approved for interventional closure of the PDA in preterm infants ≥ 700 g with promising first results [172], but further studies are necessary before a routine use can be recommended.

During the last decade, the clinical management of PDA shifted significantly from an active treatment towards “watchful waiting”, as most PDAs close spontaneously, and all interventions, pharmacological and surgical, have potential adverse effects [156,157,167,173]. A recent Cochrane meta-analysis (14 trials, *n* = 910 infants) found no difference in mortality or BPD comparing early treatment before 7 days of life versus expectant management or very early treatment before 72 h of life and versus expectant management [174].

Watchful waiting should not be interpreted as doing nothing: During “waiting”, increased positive airway pressure should be applied, and volume overload due to liberal fluid management should be avoided [175,176] (see section Fluid Management). “Watchful” means regular clinical and echocardiographic reevaluation of the PDA as recent RCTs suggest that not a binary variable “PDA existent or not” but the magnitude of PDA shunt affects the outcome [177]. Exposure to a moderate to large PDA of seven to 13 days led to an increase of the combined outcome of BPD or death (OR 2.12, 95% CI 1.04–4.32). An exposure of ≥ 14 days further increased the risk of BPD or death (OR 3.86, 95% CI 2.15–6.96) and BPD alone (OR 4.09, 95% CI 2.32–7.22) [177].

### 2.11. Fluid Management and Nutrition

The definition of restrictive or liberal fluid intake varies widely. Sung et al. proposed a very restrictive approach with 60 mL/kg on the first day of life to <116 mL/kg/day during the first weeks [175]. Other studies defined low intake as <96 mL/kg on the first day of life proceeding to <135 mL/kg/d after the first week [178]. The latter is comparable to the studies included in the Cochrane review of Bell and Acarregui [179] but exceeded that by Letshwiti et al. (mild fluid restriction 130–150 mL/kg/d) [176] or the applied cut-off value of <150 mL/kg/d in the Cochrane review of Barrington et al. [180].

The meta-analysis of early fluid management showed a reduction of hemodynamically significant PDA and NEC with a restrictive water intake, but the impact on BPD (RR 0.85, 95% CI 0.63–1.14) or mortality (RR 0.81, 95% CI 0.54–1.23) was not significant [179]. Comparable results were presented by a cohort study with an increased risk for PDA with higher fluid intake, but the association with BPD in univariate analysis remained no longer significant in regression analysis [178]. However, a retrospective secondary analysis of a RCT cohort could show the association of BPD or death with a high fluid intake and lower weight loss during the first 10 days of life [181] similar to other cohort studies [182,183,184].

Evidence on a restrictive fluid management to treat BPD is even scarcer. A Cochrane meta-analysis found insufficient data on that subject [180], with the only included study not reporting on mortality or BPD at 36 weeks PMA. The idea of reducing pulmonary edema with a resulting improvement of pulmonary function and gas exchange due to shorter diffusion distance is the rational for the widespread use of diuretics in neonatology [180,185,186]. Meta-analyses of RCTs evaluating loop diuretics as furosemide or diuretics acting on the distal tubule (thiazide and spironolactone) showed improvement in pulmonary mechanics but did not report on BPD [187,188,189]. Only two included small trials on chronic administration of thiazide-spironolactone showed a decreased mortality rate (RR 0.30, 95% CI 0.09–0.93; *n* = 77) [189]. A recent cohort study analyzed the mortality rates of infants with moderate to severe BPD in 43 US children’s hospitals. There was no difference in mortality comparing hospitals with “low” and “high” cumulative loop diuretic use [186]. Greenberg et al. examined a large cohort including 37,693 infants born before 29 weeks of gestation comparing exposure to furosemide to no exposure in regards of BPD and combined BPD or death [190]. The binary variable “exposure” yes or no was not significant, but the percentage of exposure days between the seventh day of life to 36 weeks PMA showed a reduction of BPD and of BPD or death. They could show an increase in furosemide exposure days of 10 percentage points, leading to 4.6-percentage point decrease in BPD (*p* = 0.001) and a 3.7-percentage point decrease in BPD or death (*p* = 0.01) [190]. On the other hand, a prolonged diuretic treatment with furosemide is associated with relevant side effects as electrolyte imbalances, nephrocalcinosis and ototoxicity [191].

As energy expenditure is higher in infants with BPD due to a higher respiratory rate and breathing work [192] and the association of postnatal growth failure with BPD development [193], early calorie intake likely has an important effect on BPD development. As a meta-analysis could not find any RCT evaluating standard versus high-energy intake [194], evidence for the association of BPD and lower calorie intake is based on cohort studies [184,193,195]. Especially delayed or prolonged time to full enteral feeding was associated with BPD development [183,184,193,195,196], but this may be a confounding factor, as immaturity or more critical condition led to feeding intolerance. A calorie/protein ratio below that recommended for growth was found in preterm newborns who developed BPD, and providing nutrition for these newborns remains a challenge [195]

Human milk is known to be protective against NEC [197] and seems to have a protective effect on BPD development as well. Recent meta-analyses of RCTs and observational studies found a reduction of BPD in infants fed with human milk compared to preterm formula (RR 0.78, 95% CI 0.67–0.90) [198,199]. This effect was no longer significant when only RCTs were evaluated. Another meta-analysis focused on maternal breast milk and showed a reduction of BPD in exclusively maternal breast milk-fed infants (RR 0.74, 95% CI 0.57–0.96), but as other feeding dosages with maternal breast milk showed no effect, the investigators suggested caution with the interpretation of the results [200]. Still, human milk provides various protective mechanisms, including specific nutrients and factors. However, the protein concentration may vary widely in human milk, and the fortification of human milk to prevent extrauterine growth failure is essential [201]. Standardized fortification with a fixed amount of fortifier per 100 mL of human milk is still the most applied method in NICUs. Individualized fortification, which is adjustable fortification (by measuring the infant’s blood urea nitrogen) or targeted fortification (measuring the macronutrient content of human milk), seems to be superior compared to standardized fortification regarding the gain of growth parameters in a recent Cochrane meta-analysis, but the included seven studies did not present data on the development of BPD or long-term outcomes [202]. Last, human milk has an important influence on the immature microbiome, which is an important focus of very recent research [153].

### 2.12. Vitamin A

Vitamin A supplementation is one of the prevention strategies that has stood the test of time [203] from old to new BPD, from the first RCT in 1987 [204] until the latest meta-analysis in 2021 [205]. During the fetal period, vitamin A is crucial for respiratory epithelial cells, cellular differentiation and surfactant production and vitamin A levels were found to be lower in subjects developing BPD [206], prompting the idea, that supplementation might reduce incidence or severity of BPD.

A Cochrane review showed a small risk reduction of death or oxygen requirement at 28 days of life (RR 0.93, 95% CI 0.88–0.99) and of BPD at 36 weeks PMA (RR 0.87, 95% CI 0.77–0.99, *n* = 986). However, including mortality as a combined outcome at 36 weeks PMA yields in loss of significance (RR 0.92, 95% CI 0.84–1.01) [207]. A meta-analysis on infants with a birth weight < 1000 g (ELBW) only found a decrease in BPD at 36 weeks PMA (RR 0.88, 95% CI 0.77–0.99, *n* = 1011) without an effect on mortality or oxygen use at 28 days of life [208], comparable to a more recent but smaller meta-analysis of only two RCTs [209]. Ding et al. showed in the most recent meta-analysis a reduced incidence of BPD at 36 weeks PMA (OR 0.67, 95% CI 0.52–0.88) without a difference in mortality or other morbidities [205]. However, the effect might not be big enough to justify repeated painful intramuscular injections, and it has also been questioned, whether under the circumstances of current neonatal intensive care standard therapies, the supplementation with vitamin A is still essential [210]. Two very recent RCTs analyzed enterally administered vitamin A at 5000 IU per day [211] or 10,000 IU every other day [212] and found higher plasma retinol levels in the same range of previous non-enteral studies [213]. Despite that, the Australian study found no difference in BPD or mortality in the 188 included infants born <28 weeks of gestation likely to the low baseline incidence of BPD in their cohort [211]. The Indian study of Basu et al. examined 196 VLBW infants and found a reduction of the composite outcome death or oxygen requirement at 28 days of life (RR 0.44, 95% CI 0.23–0.84) and a nonsignificant reduction of BPD at 36 weeks PMA (RR 0.22, 95% CI 0.05–1.00) [212]. A lower dose of oral vitamin A of 1500 IU per day was administered to infants born <28 weeks of gestation in a RCT for the prevention of retinopathy of prematurity (ROP) and resulted in a reduction of BPD (RR 0.56, 95% CI 0.37–0.86, *n* = 262) [214]. Two further trials are waiting for completion [215] or full publication [216]; it is to be expected that, in particular, the NeoVitaA-Trial [217] will add substantial information to the question whether there is a place for high-dose vitamin A under the circumstances of current high-standard neonatal intensive care.

### 2.13. BPD Associated Late Pulmonary Hypertension

A dysregulated angiogenesis during BPD development results in smaller pulmonary arteries and reduced capillary density. This can lead to increased pulmonary vascular resistance, followed by remodeling of the pulmonary arteries [218]. Therefore, infants with BPD are at high risk to develop pulmonary hypertension (PH). A recent meta-analysis calculated an overall prevalence of PH of 20% in preterm infants, with a distribution of 2% in the absence of BPD, 6% for mild BPD, 12% for moderate BPD and 39% for severe BPD [219], comparable to the results of an earlier meta-analysis [220]. Both studies reported on an around five-fold higher mortality of infants with BPD and PH compared to BPD alone. Furthermore, PH was associated with more frequent readmissions and a high post-discharge mortality [221,222,223]. In surviving infants, BPD-associated PH resolved in the majority of cases during the first two years of life [222,223]. PH is not uniformly defined in neonates and is usually estimated via echocardiography. Right ventricular systolic pressures (RVSP) > 40 mmHg measured via tricuspid regurgitation jet velocity (plus an estimated 5 mmHg of right atrial pressure), a RVSP/systemic systolic blood pressure ratio > 0.5, a systolic flattening of the interventricular septum or right-to-left or bidirectional cardiac shunt or PDA are the most common noninvasively obtained parameters [223,224]. The management of BPD therefore has to include an echocardiographic screening for PH, as suggested by the consensus guidelines of the European Pediatric Pulmonary Vascular Disease Network (EPPVDN) [225,226]. At least at diagnosis of BPD with 36 weeks PMA, an echocardiography should be performed to assess a possible PH [225,226,227]; other algorithms recommend serial examinations from the seventh day of life for an earlier diagnosis of PH [218]. Infants with signs of PH in a predischarge echocardiography should be referred to an outpatient cardiologic follow-up [227].

Treatment options include oxygen therapy, diuretics and pulmonary vasodilators [225]. The question of the “right” targeted SpO_2_ level in neonatal care has been the objective of many studies. A recent retrospective cohort study found a reduced incidence of PH with a higher targeted SpO_2_ of 90–95% [228]. The recommendation of EPPVDN even exceeds this threshold in infants with established PH, suggesting an oxygen saturation ≥ 93% in suspected and even ≥ 95% in proven PH [225]. At least there is robust evidence to avoid pulmonary vasoconstriction due to prolonged hypoxic episodes of SpO_2_ ≤ 85% but, also, the effect of oxygen toxicity due to hyperoxic episodes >97% [225,229].

A recent retrospective cohort study analyzed the effect of a first-line diuretic therapy in infants with BPD associated PH with substantial improvement in respiratory symptoms within one week in 90% of treated infants but without a difference in mortality or NDO [227]. It has to be mentioned that all infants in this cohort received a high total fluid intake of 155 to 158 mL/kg/d. As mentioned in Section Fluid Management, a more restrictive fluid regimen should be considered before diuretic therapy or at least after the start of diuretic therapy (thiazide plus spironolactone), as suggested by EPPVDN, as long as the cardiac preload is adequate [225]. In the case of disease progression, pulmonary vasodilator therapy should be considered after ruling out a pulmonary vein stenosis, which is often associated with premature birth [223,225,226,227,230]. Phosphodiesterase-5 inhibitors as sildenafil have been examined in retrospective cohorts, but there has been no evidence from RCTs or prospective studies exists regarding BPD-associated PH treatment with sildenafil so far. Results of a meta-analysis show an improvement in respiratory scores in 15% within two–seven days and an improvement in 69% patients regarding echocardiographic parameters of PH within one to six months without reports on serious side effects [231]. This is important, as the Food and Drug Administration (FDA) in 2012 gave an official warning on the use of sildenafil in children, especially when using higher doses [232]. However, an additional systematic review including RCTs and observational studies found the low-to-moderate dose administration of sildenafil in (older) children with pulmonary hypertension due to various causes efficient and safe [233]. With the results of both meta-analyses and following the recommendation of the EPPVDN consensus statement, the use of sildenafil (initial dose of 1 mg/kg/d titrated to a maximum dosage of 4 (to 6–8) mg/kg/d) in infants with progressive PH associated to BPD can be considered [218,225,226].

### 2.14. Stem Cell Therapy for BPD—An Outlook to a Present Future

During the last decade, stem cell therapy for the prevention and therapy of neonatal diseases has become a major research focus, with very promising results in preclinical studies [234]. Mesenchymal stem cells (MSC) are the best examined stem cell type and have shown the most potent effect on prevention or treatment of BPD in a network meta-analysis of the preclinical studies [235]. MSC are multipotent stem cells, which can be extracted from bone marrow or adipose tissue or, much easier, with a higher proliferative activity and the potential of autologous therapy from the amniotic hulls, umbilical cord or umbilical cord blood [234,236]. The regenerative mechanism is, in contrast to the first speculations, not by cell replacement (engraftment < 1–5%) but seems to depend mostly on paracrine mechanisms [234,236]. These consist of bioactive substances as signaling peptides and growths factors, extracellular matrix proteins and exosomes. The latter are membrane-bound vesicles transferring proteins and microRNA into targeted surrounding cells acting with transcriptional and posttranslational modifications in these cells [234]. This explains the evident improvement of alveolarization, angiogenesis and pulmonary arterial remodeling by cell-free therapy with MSC-conditioned media in another meta-analysis of preclinical studies [237]. One very recent study reported on the effect of MSC-conditioned media on structural and functional lung maturation, therefore targeting the lung tissue immaturity itself [238]. The network-meta-analysis still found MSC therapy superior to MSC-conditioned media regarding all four analyzed clinically relevant outcomes (alveolarization, lung angiogenesis, pulmonary hypertension and lung inflammation) [235]. All studies evaluating the safety of MSC-based therapy found no short- or long-term adverse effects, but the results have to be interpreted with caution, as almost exclusively rodent models were used in the included studies [235]. Nonetheless, in 2014, the results of the first human MSC phase I trial administering 10^7^ or 2 × 10^7^ human cord blood-derived MSCs intratracheally to nine preterm infants at high risk for BPD were published [239]. MSC therapy was well-tolerated, and a reduction in inflammatory parameters in tracheal aspirates could be shown, as well as a reduction of BPD severity compared to a historical cohort [239]. One infant died due to sepsis at the age of six months, but the two-year follow-up of this phase I trial showed no signs of transplantation-related adverse events, including tumor formation [240]. At the moment of writing the draft of this manuscript (5 February 2021), twelve recruiting or completed phase I and phase II studies evaluating the prevention or therapy of BPD with stem cells or stem cell-conditioned media have been registered at ClinicalTrials.gov, plus an additional four follow-up studies.

## 3. Recommendations for the Everyday BPD Management Based on the Presented Evidence 

In the following paragraphs, we present evidence-based recommendations for BPD management in very preterm infants. Additionally, Table 2 summarize the recommendations for BPD prevention, while Table 3 includes the recommendations for BPD therapy.

### 3.1. Antenatal Corticosteroids

ANS is an effective method in the reduction of mortality and the combined outcome of BPD or death in very preterm infants. Timing of ANS is an issue, as ANS is most effective in an interval of >24 h to seven days after the last injection. As the outcome is significantly better with an ANS course longer than seven days compared to none, ANS should be administrated in case of impending preterm birth before 34 weeks of gestation. A rescue retreatment should be considered if the first course has been completed more than one week before.

### 3.2. Surfactant-Replacement Therapy

Surfactant-replacement therapy is an essential therapy in neonatology and improved survival rates of much more immature preterm infants. Animal derived surfactant seems to be superior to synthetic surfactant, and there is weak evidence that poractant is superior to bovine surfactant. More important is the way of surfactant administration, especially in regard to the recommended noninvasive respiratory support: recent meta-analyses consistently show the superiority of LISA/MIST compared to invasive surfactant administration regarding mortality, combined BPD or death and BPD development. Therefore, this should be the preferred way of surfactant administration if the expertise of the attending physician regarding this technique is sufficient.

### 3.3. Caffeine

Based on the currently available high-level evidence from RCTs, mainly CAP, treatment with caffeine with the intention to reduce the risk of BPD can be recommended. This recommendation focuses on extremely preterm infants, since this patient group was enrolled in CAP and is also the patient group with the highest risk of developing BPD. To start caffeine treatment in the first three days of life may be considered, especially in those infants that already receive noninvasive ventilatory support, since early caffeine may help to avoid invasive ventilation in these infants.

### 3.4. Ventilation Strategies

If possible by the use of noninvasive respiratory support, mechanical ventilation should be avoided. Unfortunately, neither an optimal PEEP level for a group of infants based on pathology or patient characteristics, nor a strategy to identify the optimal level for an individual infant, could be established so far [77]. Whenever mechanical ventilation was inevitable, lung-protective ventilation strategies should be applied, and the goal should be the earliest reasonable extubation.

#### 3.4.1. High-Frequency Oscillation Ventilation

As modern respirators have evolved in terms of synchronization and tidal volume measurements, a comparison between volume-targeted ventilation modes and HFOV might be more relevant to neonatologists in the 2020s [84]. In the absence of valid recent data and with relatively small differences from meta-analyses of RCTs performed in the last four decades, it is difficult to make clear recommendations.

#### 3.4.2. Volume-Targeted-Ventilation (VTV)

Although the evidence for the most vulnerable group of ELBW infants is not as strong as for all newborn infants together, we believe it is strong enough to use VTV as the preferred mode of conventional ventilation, whenever it is possible.

#### 3.4.3. Permissive Hypercapnia

Although not supported by clear evidence, permissive hypercapnia is widely used in European NICUs [241]. While the randomized studies demonstrated difficulties in strict protocol adherence, probably because some infants require different pCO_2_ targets than those prescribed by randomization, there is no evidence to support the universal pCO_2_ target of 45 mmHg for every preterm infant. Most likely, there is a threshold beyond which intensifying the pressure, tidal volume or amplitude is increasingly harmful, and it might be wiser to let pCO_2_ increase a bit higher instead of pushing ventilator settings further and further—but we still do not know where this threshold is and if it is the same for every infant. 

#### 3.4.4. Supplemental Oxygen

Oxygen therapy in very preterm infants remains a balancing act. Oxygen saturation levels in the low 90s% should be targeted, as lower ranges (85–89%) were associated with an increased mortality [97]. In infants with suspected or proven pulmonary hypertension, the inspired fraction of oxygen should aim at higher oxygen saturation levels of 93% and 95%, respectively.

### 3.5. Tracheostomy Placement

There is insufficient evidence for the criteria and/or optimal timing of tracheostomy. Infants with persisting ventilator-dependency (mechanical ventilation or high-level noninvasive respiratory support) beyond the neonatal period might profit from a reduced sedative medication after tracheostomy and the possibility of more parent–child interaction and development supporting therapies and environment.

### 3.6. Postnatal Corticosteroids

CS have a beneficial effect on the development of BPD but are associated with severe short-term side effects and no clear-cut effect on the neurodevelopmental outcome. There is evidence that dexamethasone is more efficient than hydrocortisone, while the latter is potentially associated with lesser impact on NDO. The use of systemic CS, preferably dexamethasone, should be limited to repeated failure to extubate due to developing BPD. Increasing evidence exists for the BPD prevention with topic CS administration, yet with the unclear nonsignificant increase in mortality of NEurOSIS and paucity of long-term data after intratracheal instillation of CS, at this moment, the routine use of topic CS treatment cannot be recommended.

### 3.7. Inhaled Nitric Oxide (iNO)

There is no indication for iNO as a general prophylactic therapy to prevent BPD. It is unclear, whether there is increased risk for severe intraventricular hemorrhage for early rescue therapy, so iNO should be restricted for the most severe cases of hypoxemic respiratory failure. Only for preterm infants of African American descent, high dose prophylactic therapy should be considered.

### 3.8. Inhaled Bronchodilators

There is too little evidence to show positive or negative effects of bronchodilators for prevention of chronic lung disease, and thus, at the moment, inhaled bronchodilators should not be used routinely for this indication.

### 3.9. Macrolides

A beneficial effect of macrolides on BPD—in particular, in ventilated *Ureaplasma*-positive infants treated for a longer duration—cannot be ruled out. However, as long as this is not proven as a primary endpoint in rigorously conducted RCTs of sufficient size, and as long as safety concerns are not sufficiently addressed, we do not recommend routine treatment with macrolides.

### 3.10. Patent Ductus Arteriosus

Beside restrictive fluid intake (Section Fluid Management) and application of continuous positive airway pressure, no uniform recommendation regarding the management of the PDA can be given, as some particularly vulnerable patients may benefit from early-targeted treatment, and some infants may even profit from surgical ligation if pharmacological treatment fails. Therefore, Hamrick et al. presented a differentiated treatment algorithm considering demographic factors, echocardiographic findings and clinical assessment [157].

### 3.11. Fluid-Management and Nutrition

Moderate early restrictive fluid management in infants with hemodynamically significant PDA and/or high risk for BPD is recommended with a fluid intake of approximately 60–80 mL/kg on first day stepwise increasing by 10–20 mL/kg to a total of 130 mL/kg/d. Fluid balance, diuresis and weight loss should be carefully monitored especially during parenteral fluids are given to the infant.

To avoid unnecessary exposure to diuretics, fluid restriction should be continued in infants with signs of developing BPD as increasing need for supplemental oxygen or mean airway pressure. The physician has to weigh benefits against potential harms of a diuretic therapy. A longer treatment with thiazide plus spironolactone should only be administered if a beneficial effect on pulmonary mechanics as reduction of supplemental oxygen or mean airway pressure occurs during probatory treatment (with loop diuretics or diuretics acting on the distal tubule).

Sufficient energy supply has to be guaranteed even during reduction of water intake. To address the higher energy consumption in established BPD, a daily energy intake of >135 kcal/kg is recommended.

Early enteral feeding preferably with maternal breast milk or human donor milk may reduce development of BPD. Individualized fortification of human milk is superior to standardized fortification regarding growth parameters but there is insufficient evidence regarding BPD development.

### 3.12. Vitamin A

Taking into account the presumably small effect size and the sufficient enteral absorption, we believe it is not justified our patients to repeated intramuscular injections. Until the results of NeoVitaA tell us otherwise, we recommend supplementation of vitamin A as part of the lipid infusion in parenterally fed infants and with a water-soluble preparation in enterally fed infants in the upper range of current guidelines [242,243].

### 3.13. BPD-Associated Pulmonary Hypertension

Echocardiographic screening for BPD-associated PH should be performed at 36 weeks PMA and, eventually, in pre-discharge infants with a high risk for PH. In these infants, outpatient cardiologic follow-up is recommended. The treatment of BPD-associated PH should include higher targeted oxygen saturation and restrictive fluid management. Diuretic therapy with thiazide and spironolactone are first-line pharmacological treatments; in the case of disease progression, low-to-moderate dose sildenafil therapy should be considered.

### 3.14. Stem Cell Therapy

Despite the promising results, the administration of MSC or MSC-conditioned media for the treatment of BPD should be limited to controlled trials until efficacy and, especially, long-term safety have been proven by sufficiently powered randomized controlled trials.

## 4. Conclusions

BPD management remains an extremely challenging task, and the presented strategies for everyday work in a neonatal intensive care unit should be considered to improve the survival and outcome of very preterm infants.

## Figures and Tables

**Table 1:** Bronchopulmonary dysplasia (BPD) definition by the criteria of the National Institutes of Child Health and Human Development (NICHD)/National Heart, Lung, and Blood Institute (NHLBI) workshop of 2001 [3].

BPD Severity	Criteria
mild	Supplemental oxygen on 28th day of life and no supplemental oxygen at 36 weeks PMA
moderate	Supplemental oxygen of >21% but <30% at 36 weeks PMA
severe	Supplemental oxygen >30% or positive airway pressure or mechanical ventilation at 36 weeks PMA

PMA: postmenstrual age.

**Table 2:** Preventive strategies for BPD.

**Evidence-Based Preventive Strategies for BPD**	
Antenatal corticosteroids	Administer ANS if preterm birth < 34 weeks of GA is impending. One course of rescue-Treatment, if first course completed > 1 week ago.
Surfactant replacement therapy	Early surfactant replacement in surfactant-deficient preterm infants. LISA/MIST in combination with noninvasive ventilation is superior to surfactant with ongoing or even short invasive ventilation.
Caffeine	Early administration within the first 72 h, mainly to prevent invasive ventilation.
Ventilation strategies	Avoid invasive ventilation by the use of noninvasive respiratory support. Lung-protective ventilation: volume-targeted ventilation. Supplemental oxygen-targeting SpO_2_ levels in low 90s%, with higher thresholds only in the case of pulmonary hypertension. Aim at early extubation.
Systemic postnatal corticosteroids	Dexamethasone is more effective than hydrocortisone, but the latter might have a lesser impact on neurodevelopmental outcome. Limit systemic postnatal corticosteroid therapy to facilitate successful extubation only after repeated extubation failure.
Fluid management and nutrition	Moderate early restrictive fluid management starting from 60–80 mL/kg/d and increasing to 130 mL/kg/d. Careful monitoring of diuresis and weight loss is recommended. Human milk, especially own mother’s milk, may prevent BPD development. Provide parenteral or enteral vitamin A supplementation in the upper range of the current guidelines.
**Preventive strategies for BPD with unclear evidence**	
High-frequency oscillation ventilation	Lack of RCTs comparing volume-targeted ventilation and HFOV
Permissive hypercapnia	Unclear (and likely individually different) threshold for the targeted pCO_2_ level.
Topic corticosteroids	Inhaled CS effectively prevented BPD but with an unclear (nonsignificant) increase in mortality in the largest NEurOSIS trial.
Macrolides	Insufficient evidence for the efficiency and long-term safety for the routine use of macrolides. Treat infants with proven *Ureaplasma* spp. pneumonia with macrolides.
Patent ductus arteriosus	Apply fluid restriction and sufficient positive airway pressure to reduce the risk for relevant PDA. No uniform recommendation can be given for PDA treatment. Risk-adapted screening and treatment algorithms have been recently published.
**Preventive strategies for BPD with a lack of evidence**	
Inhaled nitric oxide	No prophylactic therapy of iNO. Infants of African-American descent might profit from high-dose prophylactic therapy. iNO should be restricted to severe hypoxemic respiratory failure with echocardiographic signs of elevated pulmonary vascular resistance.
Inhaled bronchodilators	Lack of evidence for the preventive use of inhaled bronchodilators.

*Abbreviations: ANS* antenatal corticosteroids, *GA* gestational age, *LISA* less invasive surfactant administration, *MIST* minimally invasive surfactant therapy, *BPD* bronchopulmonary dysplasia, *RCT* randomized controlled trial, *HFOV* high-frequency oscillation ventilation, *pCO_2_* partial pressure of carbon dioxide, *NEurOSIS* “Neonatal European Study of Inhaled Steroids”, *iNO* inhaled nitric oxide.

**Table 3:** Therapeutic strategies for BPD.

**Evidence-Based Therapeutic Strategies for BPD**	
Fluid management and nutrition	Continue fluid restriction in infants showing early signs of developing BPD (e.g., increasing the oxygen demand or respiratory support) or proven BPD. Higher-energy expenditure necessitates a higher caloric intake of > 135 kcal/kg/d by an increased fortification of milk.
Systemic postnatal corticosteroids	Prevention and therapy cannot be well-distinguished; therefore, the recommendation is the same as written above.
**Therapeutic strategies for BPD with unclear evidence**	
Diuretic therapy	Diuretics may improve the pulmonary mechanics. Consider long-term diuretic therapy with thiazide and spironolactone if a probatory therapy with furosemide is effective. First-line therapy in BPD-associated PH.
Stem cell therapy	Despite promising results, stem cell therapy should be limited in clinical trials until sufficient evidence for the long-term safety and effects is presented.

*Abbreviations: BPD* bronchopulmonary dysplasia, *PH* pulmonary hypertension.

## Data Availability

Not applicable, no original data were processed in this review.
